# *Allodaposuchus palustris* sp. nov. from the Upper Cretaceous of Fumanya (South-Eastern Pyrenees, Iberian Peninsula): Systematics, Palaeoecology and Palaeobiogeography of the Enigmatic Allodaposuchian Crocodylians

**DOI:** 10.1371/journal.pone.0115837

**Published:** 2014-12-31

**Authors:** Alejandro Blanco, Eduardo Puértolas-Pascual, Josep Marmi, Bernat Vila, Albert G. Sellés

**Affiliations:** 1 Institut Català de Paleontologia Miquel Crusafont, Universitat Autònoma de Barcelona, C/Escola Industrial 23, E-08201, Sabadell, Spain; 2 Grupo Aragosaurus–IUCA, Área de Paleontología, Facultad de Ciencias, Universidad de Zaragoza, Pedro Cerbuna 12, 50009, Zaragoza, Spain; University of Pennsylvania, United States of America

## Abstract

The controversial European genus *Allodaposuchus* is currently composed of two species (*A. precedens, A. subjuniperus*) and it has been traditionally considered a basal eusuchian clade of crocodylomorphs. In the present work, the new species *A. palustris* is erected on the base of cranial and postcranial remains from the lower Maastrichtian of the southern Pyrenees. Phylogenetic analyses here including both cranial and postcranial data support the hypothesis that *Allodaposuchus* is included within Crocodylia. The studied specimen suggests little change in postcranial skeleton along the evolutionary history of crocodylians, except for some bone elements such as the axis, the first caudal vertebra and the ilium. The specimen was found in an organic mudstone corresponding to a coastal wetland environment. Thus, *A. palustris* from Fumanya is the first *Allodaposuchus* reported in lacustrine-palustrine settings that expand the ecological range for this genus. The S-DIVA palaeobiogeographic reconstruction of ancestral area suggests that early members of Crocodylia rapidly widespread for the Northern Hemisphere landmasses no later than the Campanian, leading the apparition of endemic groups. In that way “Allodaposuchia” represents an endemic European clade probably originated in the Ibero-Armorican domain in the late Campanian and dispersed by the Southern European archipelago prior to the early Maastrichtian.

## Introduction

The clade of eusuchian crocodylomorphs is composed of several stem taxa and the crown Crocodylia containing the three major extant lineages: Crocodyloidea, Alligatoroidea and Gavialoidea [1]–[4]. Eusuchia probably originated in the northern continents (North America and Europe) at the beginnings of the Early Cretaceous [5]. By the Late Cretaceous, eusuchians began to diversify, a process that prolonged until the Eocene, but for some time they shared terrestrial biotas with other crocodylomorphs such as dyrosaurids, pholidosaurids, notosuchians and some relics of typical faunas of the Early Cretaceous (e.g. atoposaurids and goniopholidids) [1].

During the Late Cretaceous, eusuchians were widespread in Europe [6]. Among them, there were members of the sister taxa of Crocodylia, such as Hylaeochampsidae (e.g. the English *Hylaeochampsa vectiana* Owen, the Italians *Pietraroiasuchus ormezzanoi* Buscalioni *et al*. and *Acynodon adriaticus* Delfino *et al.*, the Iberian *Acynodon iberoccitanus* Buscalioni *et al.* and the Hungarian *Iharkutosuchus makadii* Ösi *et al.*) and the genus *Allodaposuchus* (according to Puértolas-Pascual *et al*. [5]). The crown Crocodylia was represented by the Spanish crocodyloid *Arenysuchus gascabadiolorum* Puértolas-Pascual *et al.*, and by basal alligatoroids like the French *Massaliasuchus affluvelensis* Martin & Buffetaut and the Spanish *Musturzabalsuchus buffetauti* Buscalioni *et al.* However, new phylogenetic hypotheses only recognize *Arenysuchus* and *Musturzabalsuchus* as closely related to the crown group [5], [6]. Hence, current classification should be revised as more complete specimens are recovered. On the other hand, several species of basal gavialoids attributed to the genus *Thoracosaurus* have been described from the Late Cretaceous of France, Netherlands and Crimea [6].

The systematic emplacement of some basal eusuchians is controversial, especially due to their fragmentary nature and the lack of postcranial remains for the majority of species. This is particularly true of the European genus *Allodaposuchus*. For instance, while some analyses place it as a sister taxon of Crocodylia [5]–[8], others suggest that it was related to the clade Alligatoroidea + Crocodyloidea [4] or included within Alligatoroidea [9]. Nopcsa [10] erected the genus *Allodaposuchus*, and its type species *A. precedens*, on the basis of some cranial and limited postcranial material from Vălioara (Haţeg Basin, Romania) and Valdonne (Fuveau Basin, France) localities. Later on, Buscalioni *et al.* [7] performed a revision of these materials and included new fragmentary remains from Spain and France, based on characters widely distributed in basal eusuchians. Subsequently, Martin and Buffetaut [11], after reviewing the French material from Valdonne, erected the new taxon *Massaliasuchus affuvelensis*. More recently, Delfino *et al*. [8] described a new complete skull from Oarda de Jos (Romania) that was ascribed to *Allodaposuchus precedens*, and also argued that specimens from Western Europe should be considered as a different taxon from *A. precedens*. Finally, Puértolas-Pascual *et al*. [5] erected *Allodaposuchus subjuniperus* on the base of a nearly complete skull from Huesca (NE Spain). Accordingly, nowadays the genus *Allodaposuchus* includes two formal species, the Romanian *A. precedens* Nopcsa and the Spanish *A. subjuniperus* Puértolas-Pascual *et al.*, and other remains with ambiguous attribution at species level.

**Figure 1:**
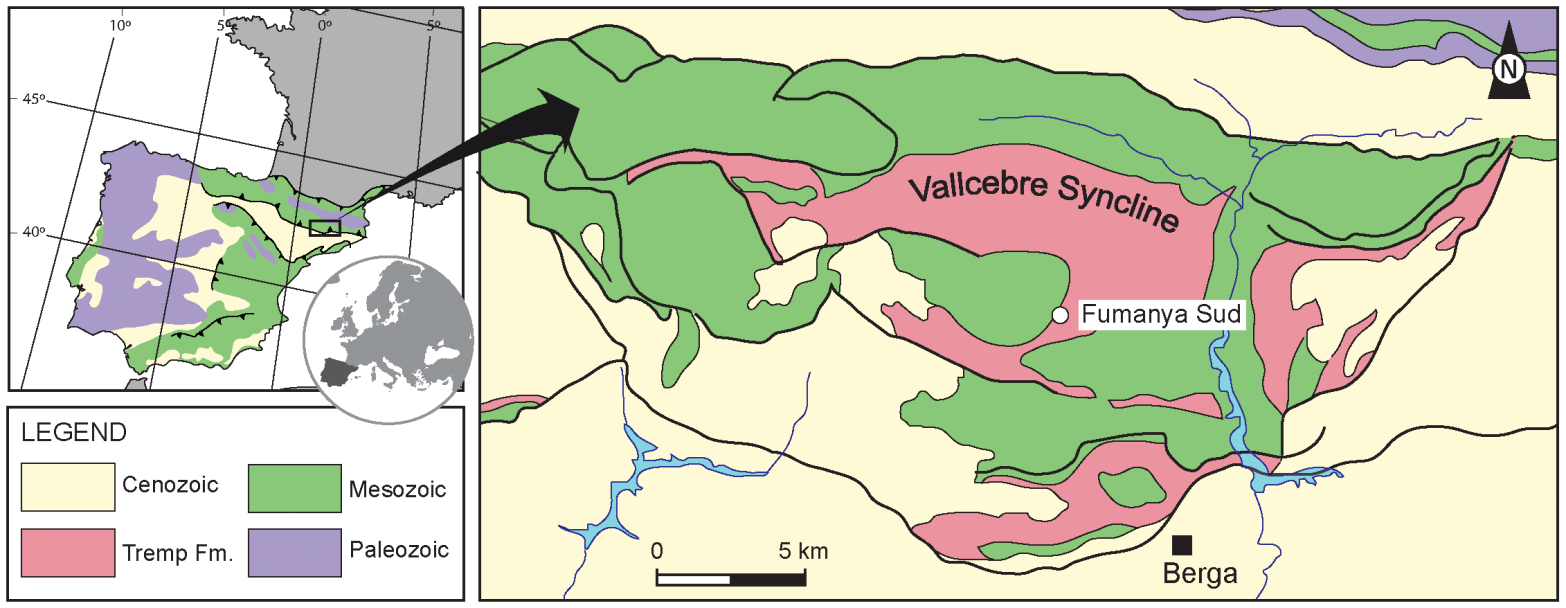
Geological setting of the Fumanya Sud locality within the Iberian Peninsula.

In this paper, we describe new remains of an eusuchian crocodylomorph found in the Tremp Formation beds exposed at the Fumanya Sud locality (lower Maastrichtian, northeastern Iberian Peninsula, Fig. 1). They are attributed to the genus *Allodaposuchus* and consist of partial skull and numerous postcranial elements belonging to both axial and appendicular skeletons. Thus, it represents the most complete postcranial material known for *Allodaposuchus* so far and it is relevant to understanding the evolution of postcranial skeleton in eusuchians. The aims of the work are: 1) describe the postcranial skeleton of *Allodaposuchus* and compare it with other stem and crown eusuchians, 2) enhance the phylogeny of basal eusuchians and, especially, shed light on the conflicting phylogenetic relationships of the genus *Allodaposuchus*, 3) describe the habitat and palaeoecology of the new specimen, and 4) provide new data on the species diversity and palaeobiogeography of Late Cretaceous eusuchians in Europe.

### Geological setting

The Tremp Formation [12] consists of transitional and continental materials deposited in an E–W foreland trough and following a marine regression that began near the Campanian–Maastrichtian boundary [13]. After the uplift of the Pyrenean range, these deposits were separated into four main synclines which are, from the east to west: Vallcebre, Coll de Nargó, Tremp and Àger. In the south-central and south-eastern Pyrenees, Rosell *et al*. [14] divided the Tremp Formation, or the so-called ‘Garumnian’ facies [15], into four lithologic units, which are from the base to the top: i) a transitional 'grey unit' (marls, coals, limestones, and sandstones), ii) a fluvial 'lower red unit' (mudstones, sandstones, oncoids, and paleosols), iii) the lacustrine 'Vallcebre limestone and laterally equivalent strata' and, iv) a fluvial 'upper red unit' (mudstones, sandstones, conglomerates and limestones). At the Vallcebre Syncline, the two former units are Maastrichtian in age whereas the two later are Paleocene, according to charophyte biostratigraphy [16] and magnetostratigraphy [13].

**Figure 2:**
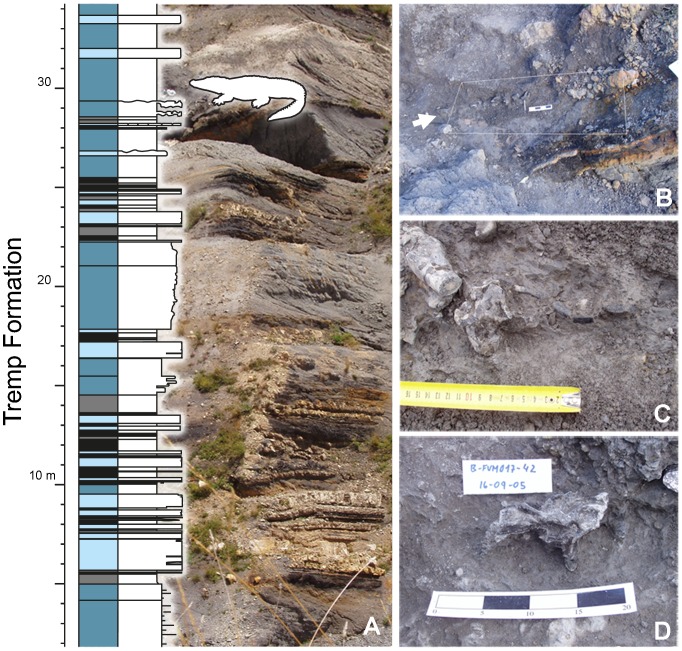
Stratigraphic location and field aspect of the crocodilian fossils at the Fumanya Sud locality. A, stratigraphic log and outcrop view of the sequence of alternating dark mudstones, limestones and lignites in the 'grey unit' with indication of the bone-bearing horizon. B, metric grid used to mapping the fossils along a digging transect of about 2.5 m long and 30 cm thick. C, field aspect of a lumbar vertebra, a partial tibia and some rib fragments. D, Dorsal vertebra (D5) preserving the centrum and parts of the apophyses in connection with the neural arch, as found in the outcrop. Scale bars in centimeters.

In the Fumanya Sud locality (Vallcebre Syncline), the studied specimen was found approximately 30 meters above the base of the Tremp Formation within a 45 meter thick sequence of alternating dark mudstones, limestones and lignites (Figs. 1 and 2). The sequence is included in the 'grey unit' of Rosell *et al.* [14], more specifically within the 'middle grey garumnian' of Villalba-Breva *et al.* [17]. The sedimentological analysis and the palaeontological content (charophytes, coals, rooting structures and brackish to freshwater mollusks) of this part of the sequence suggest a lacustrine-palustrine environment [17], as part of a more extensive lagoon [13], [14], [18]. Magnetostratigraphy indicates an early Maastrichtian age for the site, within the C31r [13].

The crocodylomorph fossils occurred at the lower part of a dark organic-matter-rich mudstone (about 40 centimeters in thickness. Fig. 2A). It corresponds to the F2 facies of Oms *et al.* [13] and overlays a 10-cm thick limestone with synsedimentary microkarstification that evidences probable sporadic subaerial exposure. Forty centimeters below the limestone there is a 10-cm thick black mudstone, usually mistaken with coal. The absence of root marks suggests parautochthonous accumulation of organic matter in coals overlaying black mudstones.

## Materials and Methods

### Studied material

The studied material consists in a disarticulated specimen (MMC829 - MMC894, B-FUM017-62, B-FUM107-71) housed at the Museu de les Mines de Cercs (Sant Corneli, Barcelona, Spain). The Departament de Cultura de la Generalitat de Catalunya issued the permission for the studied locality. All necessary permits were obtained for the described study, which complied with all relevant regulations.

The specimen herein described was discovered by one of the authors (B.V.) during prospecting works conducted in 2004 at the Fumanya Sud locality and was excavated during three subsequent field campaigns, between 2004 and 2008, under the permission of the Government of Catalonia. Small hand tools facilitated exposure of the bones that were then consolidated and mapped using a metric grid and graph paper, within a digging transect of about 2.5 m long and 30 cm thick (Fig. 2B). Final works at the site consisted in the removal of the contiguous upper sediments around the bone accumulation in order to rule out the presence of more fossils at deeper levels. During the fieldwork, a stratigraphic section was drawn for a 1.5 m-thick interval that contained the crocodylomorph bones. After preparation, crocodylomorph fossils were housed at the Museu de les Mines de Cercs.

Morphological characters of the Fumanya specimen were compared with basal eusuchians (*Isisfordia duncani*, in Salisbury *et al.* [19]), hylaeochampsids (*Pietraroiasuchus ormezzanoi*, in Buscalioni *et al.* [2]), related species of genus *Allodaposuchus* (*A. precedens* [8]; *A. subjuniperus* [5]) and *Arenysuchus gascabadiolorum* [4], gavialoids (*Gavialis gangeticus* Gmelin, in Brochu [20]), basal alligatoroids (*Diplocynodon hantoniensis* Wood, in Brochu [20]; *Musturzabalsuchus buffetauti*, in Buscalioni *et al.* [21], *Massaliasuchus affluvelensis* [11] and crocodyloids (*Crocodylus acutus* Cuvier, in Mook [22]). In addition, postcranial skeletons of extant crocodyloids (*Crocodylus niloticus* Laurenti (MZB 2003-1423), *Osteolaemus tetraspis* Cope (MZB 2006-0039) and alligatoroids (*Alligator mississipiensis* Daudin, MZB 92-0231, MZB 2006-0613), housed at the collection of the Museu de Ciències Naturals de Barcelona, were used for comparisons and measurements.

### Cladistic analysis

Phylogenetic relationships of the specimen from Fumanya were explored using the dataset of Brochu [3]. However modifications in some operational taxonomic units (OTUs) and characters were carried out (see S1 Information).

The whole dataset resulted in 85 OTUs, which were coded for a total of 181 craniodental and postcranial characters. The taxon *Bernissartia fagesii* Dollo was used as outgroup. Two matrices were used for analyses. The first one included both craniodental and postcranial characters and the second one included craniodental characters only (see S2 and S3 Information). Datasets were analysed with TNT v1.1 (Willi Hennig Society Edition, [23]). Tree-space was searched using a heuristic search algorithm (traditional search method) with tree-bisection-reconnection branch swapping and 1,000 random addition replicates holding 10 most parsimonious trees for each replicate. All characters were equally weighted and multistate characters were unordered. Bremer supports and bootstrap frequencies (1,000 bootstrap replicates searched) were used to assess the robustness of the nodes.

### Palaeobiogeographic analysis

In recent times, the development of mathematical models to infer the ancestral area of origin for a certain taxonomic group has improved our understanding on how speciation patterns work in both present and past times. These types of biogeographical analyses rest upon phylogenetic relationships, known geographical distribution of the taxa, and their time-range. In order to explore the historical palaeobiogeography of Crocodylia and especially to establish the role that *Allodaposuchus* played in the early radiation of the group, we reconstruct the ancestral area by using the Statistical Divergence-Variance methodology (S-DIVA) developed by Yu *et al.* [24]. The phylogenetic matrix employed in the phylogenetic study was implemented in RASP 2.1 software [25]. Geographic distribution of extant and fossil taxa used in the analysis, which was gathered from the literature, was established according to major continental landmasses, those including Europe, North America, South America, Asia, Africa, and Australia. Combination of two geographically close related areas (i.e., North America + Europe or Asia + Europe) was also considered for the present analysis.

### Nomenclatural Acts

The electronic edition of this article conforms to the requirements of the amended International Code of Zoological Nomenclature (ICZN), and hence the new names contained herein are available under that Code from the electronic edition of this article. This published work and the nomenclatural acts it contains have been registered in ZooBank, the online registration system for the ICZN. The ZooBank LSIDs (Life Science Identifiers) can be resolved and the associated information viewed through any standard web browser by appending the LSID to the prefix "http://zoobank.org/". The LSID for this publication is: urn:lsid:zoobank.org:pub: 74D0FF1E-4095-4E07-A11A-31DFCE17B51E. The electronic edition of this work was published in a journal with an ISSN, and has been archived and is available from the following digital repositories: PubMed Central, LOCKSS.

## Systematic Palaeontology

Superorder CROCODYLOMORPHA

Order CROCODYLIFORMES Hay, (*sensu* Benton and Clark)

Suborder EUSUCHIA Huxley,

Unranked CROCODYLIA Gmelin, (*sensu* Benton and Clark)

Genus *Allodaposuchus* Nopcsa,

### Emended diagnosis for the genus

*Allodaposuchus* differs from all other eusuchians by the exclusive combination of the following synapomorphies: margin of the orbits upturned; quadrate and squamosal not in contact on the external surface of the skull, posteriorly to the external auditory meatus; caudal margin of otic aperture not defined and gradually merging into the exoccipital; dermal bones roof overhang rim of supretemporal fenestra; cranioquadrate passage or canalis quadratosquamosoexoccipitalis laterally open and represented by a sulcus (broader than in *Hylaeochampsa vectiana* [26]), with the exoccipital between the squamosal and the quadrate posterior to otic aperture. Ventral process of the exoccipital not involved in the basioccipital tubera; quadrate foramen aereum on dorsal surface.

### *Allodaposuchus palustris* sp. nov

(Figs. 3–7)

urn:lsid:zoobank.org:act:EA300B82-1108-4B42-9C4C-99A2A9D496C8

**Figure 3:**
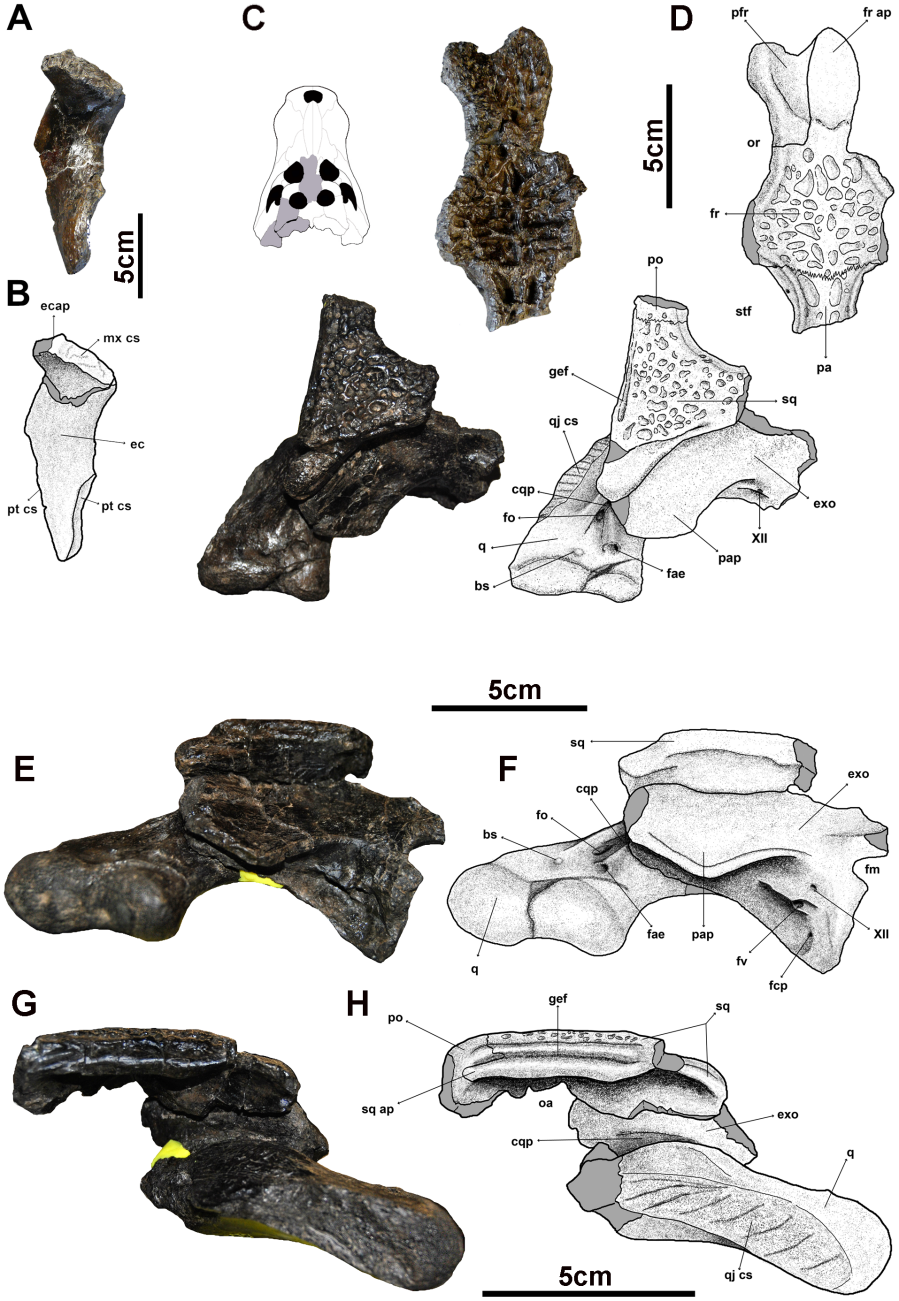
Cranial remains of *Allodaposuchus palustris* sp. nov. from the Fumanya locality. A, ectopterygoid; B, interpretative drawing of A; C, reconstruction of the skull table and left posterior part of the skull in dorsal view with original bones (marked in grey in the small skull drawing); D, interpretative drawing of C; E, squamosal, exoccipital and quadrate in posterior view; F, interpretative drawing of E; G, H, the same bones and interpretative drawing in lateral view. Abbreviations: XII, foramen for cranial nerve XII; bs, boss; cqp, canalis quadratosquamosoexoccipitalis; ec, ectopterygoid; ecap, ectopterygoid anterior process; exo, exoccipital; fae, foramen aërum; fcp, carotid foramen; fm, foramen magnum; fo, foramen; fr, frontal; fr ap, frontal anterior process; fv, foramen vagi; gef, groove for ear flap; mx cs, maxillar scar; oa, otic aperture; or, orbit; pa, parietal; pap, paropcipital process; pt cs, pterygoid scar; pfr, prefrontal; po, postorbital; q, quadrate; qj cs, quadratojugal contact surface; sq, squamosal; sq ap, anterior process; stf, supratemporal fenestra.

**Figure 4:**
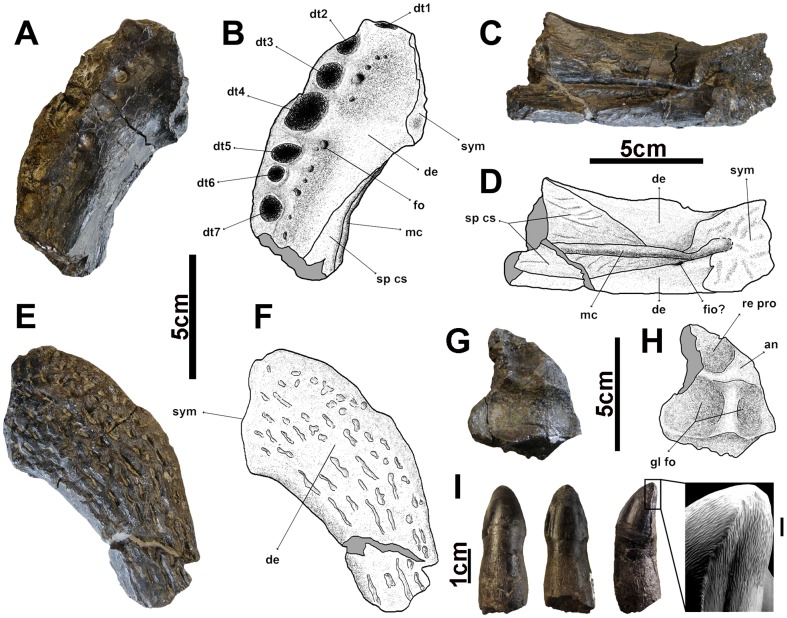
Mandibular bones and teeth of *Allodaposuchus palustris* sp. nov. from the Fumanya locality. A, C, E, anterior tip of the left dentary in occlusal, lingual and ventral views; B, D, F, interpretative drawings of A, C and E; G, articular; H, interpretative drawing of G; I, a tooth in labial, lingual and lateral views and a detail of the ornamentation of the crown surface (scale 1 mm). Abbreviations: an, angular; dt1-7, tooth alveoli1-7; de, dentary; fim, foramen intermandibularis oralis; fo, foramen; gl fo, glenoid fossa; mc, Meckelian canal; re pro, retroarticular process; sp cs, splenial scar; sym, symphysis.

**Figure 5:**
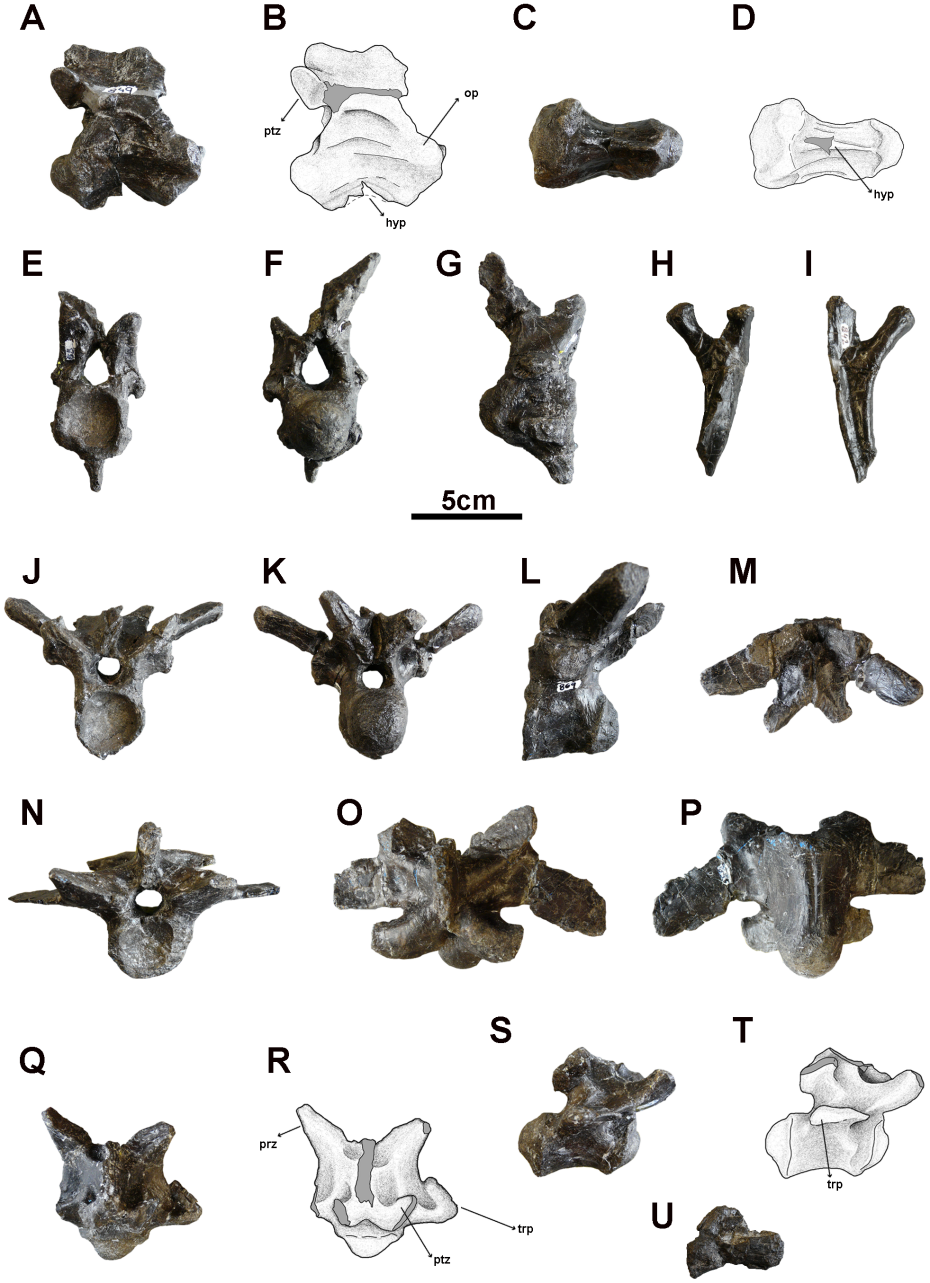
Axial skeleton bones of *Allodaposuchus palustris* sp. nov. from the Fumanya locality. A, left side of the axis; B, interpretative drawing of A; C, ventral view of the axis; D, interpretative drawing of C; E–G, 7^th^ cervical in anterior, posterior and lateral views; H–I, proximal part of the eighth cervical rib in lateral and medial views; J–M, 5^th^ dorsal vertebra in anterior, posterior, lateral and dorsal views; N–P, 2^nd^ lumbar vertebra in anterior, dorsal and ventral views; Q, S; first caudal vertebra in dorsal and lateral view; R, T, interpretative drawings of Q and S; U, undetermined caudal vertebra. Abbreviations: hyp, hypapophysis; op, odontoid process; prz, prezygapophysis; ptz, postzygapophysis; trp, transverse process.

**Figure 6:**
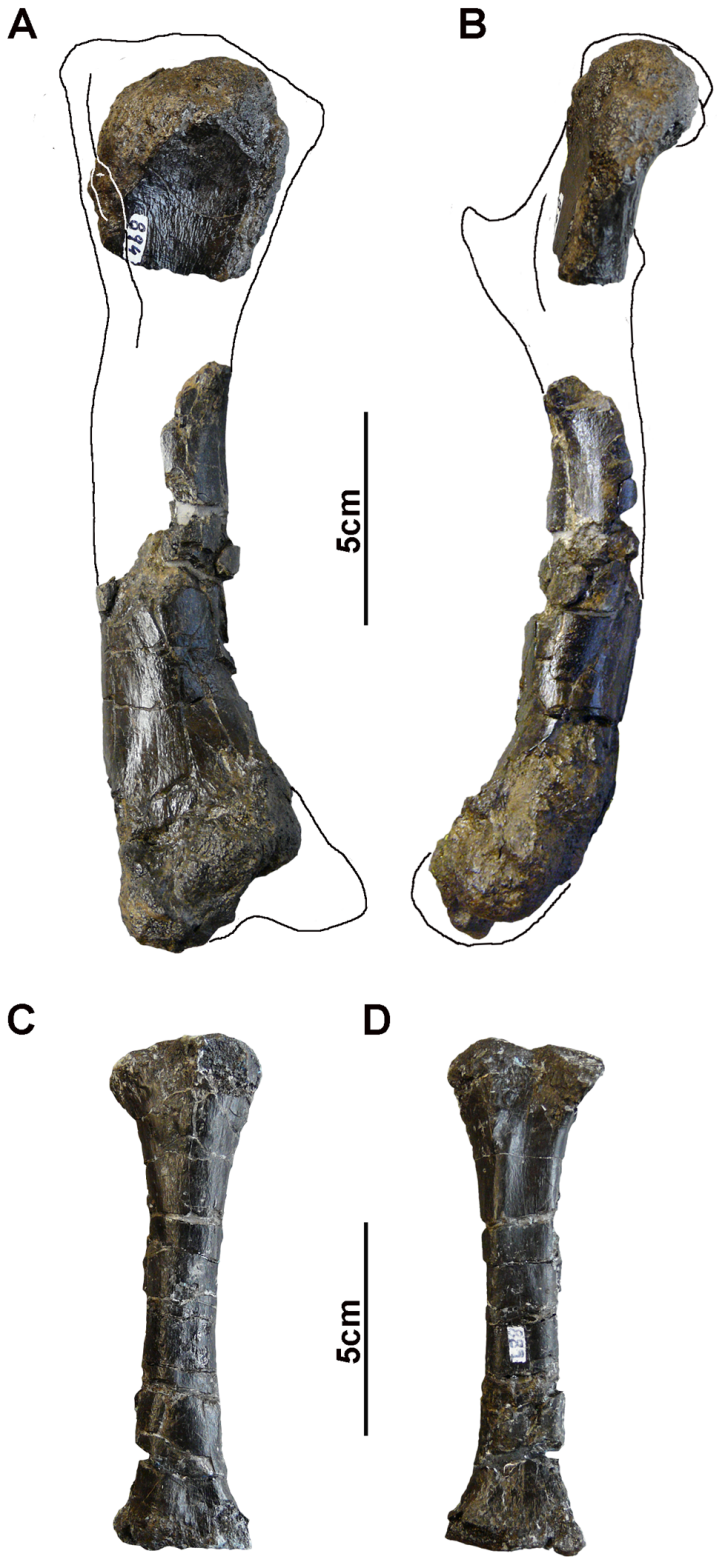
Forelimb bones of *Allodaposuchus palustris* sp. nov. from the Fumanya locality. A–B, humerus in medial and frontal views; C–D; radius in lateral and medial views.

**Figure 7:**
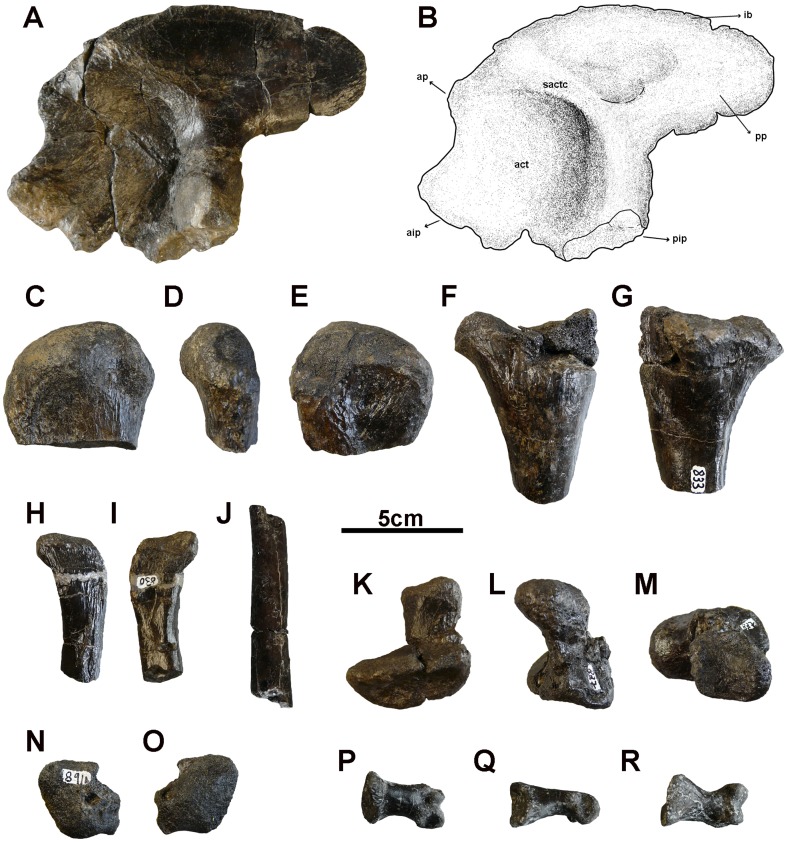
Pelvic scar and hindlimb bones of *Allodaposuchus palustris* sp. nov. from the Fumanya locality. A, left ilium in lateral view; B, interpretative drawing of A; C–E, proximal epiphysis of the left femur in anterior, lateral and posterior views; F–G, proximal epiphysis of the left tibia in anterior and posterior views; H–J, proximal fragment and diaphysis of the left fibula; K–M, calcaneum in distal, lateral and anterior views; N–O, astragalus in anterior and posterior views; P–R, undetermined phalanx in dorsal, lateral and ventral view. Abbreviations: act, acetabulum; aip, anterior ischiadic process; ap, anterior process; ib, iliac blade; pip, posterior ischiadic process; pp, posterior process; sactc, supracetabular crest.

urn:lsid:zoobank.org:act:EA300B82-1108-4B42-9C4C-99A2A9D496C8

#### Etimology

*palustris*, from Latin “palus”, swamp.

#### Diagnosis

Lack of shallow fossa in the rostromedial margin of the supratemporal fenestra; frontoparietal suture slightly concavoconvex; exoccipital without boss on paraoccipital process; large foramen aereun in quadrate; short and robust teeth with two very marked longitudinal grooves close to the carinae; teeth with strong ornamentation developing false-ziphodont crenulations; anterior process of the ilium more developed.

Ambiguous autapomorphies: neural spine of the axis with a medial depression; keel of the axis occupies completely the ventral side of the centrum; iliac blade with very rounded and wrinkled superior border, and elevated posterior process; large distal end of the calcaneum. We prefer coding all these autapomorphies as ambiguous, due to the absence of postcranial remains in other species of *Allodaposuchus*. New discoveries may reveal if they are autapomorphies of the genus.

#### Holotype

The holotype is a partial skeleton (housed in the Museu de les Mines de Cercs, Barcelona) that comprises 9 isolated teeth (MMC-829a, MMC-829b, MMC-846, MMC-850, MMC-851, MMC-854, MMC-856, MMC-879, FUM017-62), one fragmentary dentary (MMC-857, MMC-870, MMC-876, MMC-881), left prefrontal and fused frontals (MMC-892), left squamosal (MMC-859), left ectopterygoid (MMC-864), left quadrate (MMC-862), 2 articulars (MMC-834, MMC-871), left exoccipital (MMC-866), 4 cervical vertebrae (MMC-849, MMC-865, MMC-875, MMC-883), 7 dorsal vertebrae (MMC-839, MMC-853, MMC-869, MMC-872, MMC-873, MMC-882, MMC-890), 2 lumbar vertebrae (MMC-835, MMC-888), 3 caudal vertebrae (MMC-843, MMC-886, FUM017-71), 2 well preserved ribs (MMC-840, MMC-842), one right humerus (MMC-861, MMC-894), one left radius (MMC-889), one left ilium (MMC-838), one fragment of the left femur (MMC-863), a fragmentary left tibia (MMC-833), left fibula fragments (MMC-830, MMC-844), left calcaneum (MMC-837), left astragalus (MMC-891), and two phalanges (MMC-832 MMC-844).

#### Locality, age and horizon

Fumanya Sud (North Barcelona, Catalonia), early Maastrichtian (C31r) as determined by charophyte biostratigraphy and magnetostratigraphy [13]. The fossil-bearing horizon is found 29 meters above the top of “concrete level”, the basis of the Tremp Formation [13].

## Description

### Cranial skeleton

The cranial remains were scarce, fragmentary and disarticulated (Fig. 3 and 4). Nevertheless, some identifiable elements have been preserved such as the left anterior region of the dentary and other undetermined parts of the dentary, the left and right articular, several isolated teeth, the left ectopterygoid, the frontal, part of the left prefrontal, the left squamosal, the left exoccipital and the left quadrate. Despite being isolated, these cranial elements, allowed a detailed description of each one and the main morphology of the skull table, left occipital region and part of the skull openings.

#### Cranial openings

All the medial rim of the left orbit is preserved being able to interpret its general morphology (Fig. 3C). The orbits are relatively large, rounded and slightly elongated rostrocaudally with their rostromedial margin somewhat elevated. In lateral view, the prefrontal-frontal suture in the medial wall of the orbit is vertical. There are several foramina in the medial wall of the orbit, the largest is placed in the prefrontal and there are five smaller foramina rostrocaudally aligned in the frontal.

Only the rostromedial and caudolateral margins of the left supratemporal fenestra are preserved (Fig. 3C). The supratemporal fenestra is circular and smaller than the orbits. The skull roof overhangs the fenestra in the medial and caudolateral rims. There is no shallow fossa forming a step or notch in the rostromedial margin of the supratemporal fenestra, being this wall almost vertical. There are two foramina in the rostromedial and medial wall of the supratemporal fenestrae, one is located on the frontoparietal suture and the second one is placed in the parietal.

The quadrate, the squamosal and the exoccipital are fully preserved and it is possible to reconstruct the partial morphology of some structures related with the otic region (Fig. 3C–H). The otic aperture does not have a sharp posterior rim, and the cranioquadrate passage forms a caudolaterally open sulcus called canalis quadratosquamosoexoccipitalis [7], [8], [27]. The squamosal and the quadrate are not in contact posteriorly to the otic opening, being the exoccipital between these two bones (Fig. 3G–H).

#### Ectopterygoid

The left ectopterygoid (MMC-864) is fully preserved (Fig. 3A–B). It is very broad and robust, with the anterior process strongly ventrolaterally twisted. However, this region is partially broken and glued, and this torsion may be a deformation effect. Because the ectopterygoid was recovered isolated, the exact contact relationships with the maxilla and the pterygoid are unknown, and the overall shape of the suborbital fenestra cannot be interpreted.

#### Frontal

The frontals are fused into a whole single bone (MMC-892) and part of the left prefrontal is also preserved (Fig. 3C–D). The anterior process of the frontal is separated from its main body approximately in the middle of the medial margin of the orbits. This anterior process is heavily ornamented with pits and grooves and it is lanceolate in shape being probably shorter than the prefrontal. The preserved part of the prefrontal is upturned forming a transverse ridge in the rostromedial margin of the orbits. The main body of the frontal is slightly concave. The frontal forms the caudomedial corners of the orbits and part of the rostromedial edges of the supratemporal fenestrae. The frontoparietal suture is slightly concavo-convex and enters the rostromedial margins of the supratemporal fenestrae.

#### Squamosal

Only the left squamosal (MMC-859) has been preserved and it is almost complete (Fig. 3C–D). The squamosal forms the caudolateral margin of the supratemporal fenestra overhanging it. Its dorsal surface is flat, horizontal and fully ornamented with circular pits. The lateral margins of the skull table appear to be straight rather than curved or convex. The posterior region is formed by a squamosal prong that projects caudoventrally to contact the exoccipital, the squamosal and the quadrate are not in contact in this region (Fig. 3E–H). This prong lacks ornamentation and is not laterally projected. In lateral view there are two nearly parallel rims that form the groove for the attachment of musculature for the ear valve (Fig. 3G–H). The squamosal has a lobular anterior process that extends ventrally to the caudodorsal end of the postorbital (Fig. 3G–H).

#### Quadrate

The left quadrate (MMC-862) is almost fully preserved (Fig. 3C–H). The surface of the quadrate is smooth, without ornamentation. Its dorsal surface has a sulcus caudolaterally directed from the otic aperture that forms the ventral margin of the canalis quadratosquamosoexoccipitalis. This canalis is laterally delimited by a marked crest and the medial margin of the canalis is delimited by the exoccipital (Fig. 3E–H). At the end and within the canalis there is a foramen, and posterior to this foramen there is a marked circular boss (Fig. 3D, F). Medially to the foramen and boss, there is a large foramen aërum on the dorsal surface of the quadrate (Fig. 3D, F). Both quadrate hemicondyles are slightly dorsoventrally expanded. The lateral hemicondyle is larger, and both hemicondyles are rounded (Fig. 3C–F). The ventral surface of the quadrate is very smooth without well-marked crests.

#### Exoccipital

Only the left exoccipital (MMC-866) has been preserved (Fig. 3E–F). The ventrolateral region of the paroccipital process forms a well-marked crest caudoventrally directed, but there is no boss on paroccipital process. The paroccipital process is short and just slightly surpasses laterally the caudolateral aperture of the canalis quadratosquamosoexoccipitalis, which remains laterally opened. In posterior view, although the basioccipital is not preserved, it seems that the ventral process of the exoccipital is short, smooth and not involved in the basioccipital tubera. This is due to none rugose posterior expansion is observed in this region (Fig. 3E–F). Below the paroccipital process there is a concave region crossed by ridges that surround the large foramen vagi. The foramen for the XII cranial nerve and the carotid foramen are smaller and difficult to distinguish.

#### Mandible

The most anterior tip of the left dentary (MMC-876) has been preserved. Furthermore, undetermined parts of the mandible (MMC-857, 870, 881) and part of the left (MMC-871) and right articulars (MMC-834) have also been recovered. The anterior region of the dentary has the alveolar margin at the same dorsal height. The first seven alveoli of the dentary are preserved (Fig. 4A–B) and they are slightly dorsolaterally projected. The largest tooth alveolus is the fourth, being almost twice as large as the other preserved alveoli. The ventrolateral surface of the dentary (Fig. 4E–F) is densely vascularized with elongated grooves. The dentary symphysis is short and extends back to the level of the fourth dentary alveolus (Fig. 4A–D). The dorsomedial surface of the dentary is broad and smooth between the symphyseal surface and the tooth row. Medially to the tooth alveoli, the dentary is strongly vascularized with small and aligned foramina (Fig. 4A–B). The splenial is not preserved, but its attachment scar on the medial surface of the dentary can be observed. The splenial does not enter in the symphysis and its anterior tip is ventrally placed to the Meckelian canal (Fig. 4C–D). There is a foramen in the anterior most tip of the splenial-dentary contact, but we cannot know if the splenial was perforated by the intermadibularis oralis foramen. The left and right articulars have been partially preserved but they provide little information due to their fragmentary nature (Fig. 4G–H).

#### Dentition

Several isolated teeth have been preserved (MMC-829, 846, 850, 851, 854, 856, 879, and FUM017-62). All teeth have a similar morphology and only vary in size and elongation degree. The teeth are conical to lanceolate, relatively short and robust, with the labial surface more convex than the lingual surface (Fig. 4I). They show a slight constriction in the base. The carinae are well developed in the anterior and posterior views of the teeth. In the lingual surface two very marked longitudinal grooves close to the carinae are developed. The teeth have strong ornamentation composed of numerous and fine longitudinal ridges, conspicuous to the apex, that present a strong anastomosis that crosses the carinae developing false-ziphodont crenulations.

### Axial skeleton

#### Axis

The axis (MMC-849) is a characteristic vertebra (Fig. 5A–B). The neural arch is low. It is 4.8 cm high at its posterior end and the spine is low and elongated craniocaudally. The anterior border of the spine is slightly rugose and slightly thickened compared to the posterior one. At its middle part, the spine makes a small depression, which is concave in lateral view. The spine projects caudally beyond the postzygapophyses. The anterior end of the neural arch is broken and the shape of the prezygapophyses is not clearly recognisable. The postzygapophyses face obliquely ventrolaterally. Tubercular and capitular facets are located in the odontoid process (Fig. 5A). The neural arch and the odontoid process are completely sutured to the centrum, suggesting an adult specimen. The centrum is 7.05 cm long and ventrally keeled. The keel (hypapophysis) is a thin layer and completely occupies the ventral side of the centrum (Fig. 5C–D), which is slightly convex ventrally in lateral view forming an obtuse angle of 145°.

#### Remaining cervical vertebrae

The third (MMC-875), fifth (MMC-865) and seventh (MMC-883) (Fig. 5E–G) cervicals are also preserved. They are higher than wide. The neural spine is lost in all the three vertebrae. Along the cervical series, the diapophyses are progressively oriented from ventral to perpendicular to the sagital plan. Therefore, the tubercular facets, located in the diapophyses, face ventrolaterally in the third and fifth cervicals, whereas are nearly flat in the seventh cervical. Zygapophyses are oriented craniocaudally, nearly parallels to the longitudinal axis. The centra are procoelous and cranially keeled in the ventral side, but the keel is only completely preserved in MMC-883 (Fig. 5E–G). Their lengths are similar (∼4.7 cm) along the series, but their diameters increase progressively from 3.0 to 3.7 cm. In the three vertebrae, the capitular facets are located in the parapophyses, in a lower position within the centrum.

#### Dorsal vertebrae

The preserved dorsal vertebrae have been sorted based on the presence/absence of keels as well as their shape, and the location of the parapophyses. The first, third and fifth dorsal vertebrae (MMC-873, 872 and 869 respectively) and other four dorsals of uncertain position (MMC-853, MMC-882, MMC-839, MMC-890) have been recovered. The shape of the first and third dorsals resembles that of the cervicals. The spines are relatively high, the keel is prominent, and the capitular facets are located in the parapohyses, but in an upper position within the centrum. The fifth dorsal (Fig. 5J–M) is not much different, but it presents a vestigial keel. It also has lost the parapophyseal processes and the capitular facets are located at the base of diapophyses (Fig. 5L). The neural arch and spine seem to progressively increase in high and bend caudally. It is about 4.8 cm high in D1 but incomplete in the others. Diapophyses also increase in size caudally (7.9 to 10.8 cm in D1 and D5, respectively). Zygapophyses are broader than those of the cervicals and dorsomedially oriented. The centra are procoelous and similar in length (∼5 cm) and diameter (∼ 3.7 cm) to the last cervical.

The last four vertebrae are too eroded to perform a detailed description. They are believed to be equal or posterior to the sixth dorsal position, based on the morphology of the diapophyses, the position of the parapophyses and the absence of the keel. Some of them preserve a part of the neural arch and the postzygapophyses, but none is complete. Zygapophyses are broader than those from the anterior dorsals and cervicals, and are laterally oriented. Neither the parapophyses nor the keel are present in the centrum. In addition, the centra are slightly longer (up to 5.8 cm) than those from the anterior dorsals.

#### Lumbar vertebrae

The first (MMC-888) and second (MMC-835) lumbar vertebrae are present (Fig. 5N–P). These two lumbar vertebrae are similar in shape, but L1 is slightly smaller in size. They are slightly eroded. The neural spines are craniocaudally wide and lower (1.6 cm high) than those of the dorsals and cervicals. Transverse processes and zygapophyses are elongated and broad (∼12.5 cm wide in L2). The centra are procoelous and longer (∼7.5 cm in L2) than those of the anterior vertebrae. The L2 has a longitudinal groove ventral to the centrum (Fig. 5P).

#### Caudal vertebrae

Only three caudal vertebrae have been recovered (MMC-843, MMC-886, FUM017-71). The left transverse process, postzygapophyses and the end of the neural spine are not preserved in the largest caudal (MMC-843, Fig. 5Q–T). Its right transverse process is very short (1.5 cm long) and thick (Fig. 5Q–R). Prezygapophyses are oriented craniolaterally, and their articular surfaces are wide, long and face medially. MMC-843 is identified as the first caudal vertebrae based on the biconvex centrum, unique through the complete axial skeleton of eusuchians (Fig. 5S–T). The centrum is similar in size (∼7 cm long) to the lumbars. The MMC-886 is interpreted as a middle to posterior caudal vertebra (Fig. 5U). No transverse process is present and the lateral surface is smooth. The centrum is elongated, 4.6 cm long, and the neural spine is broken and eroded. The FUM017-71 is a more posterior caudal vertebra. It is similar in shape to MMC-886, but smaller in size (4.4 cm long).

#### Ribs

MMC-842 and MMC-840 are C8 and D1 left ribs, respectively. The ribs of the eighth cervical are intermediate in form between the cervical and dorsal ribs (Fig. 5H–I). The anterior process of the shaft of a typical cervical rib is present in the rib of C8. The process is thin, extends cranially from the shaft, below the capitulum and tuberculum. These two articular processes are similar in length, width and angle respect to the shaft, making a symmetrical shape for the proximal part of the bone. The shaft is flattened, and presents a long groove in the ventral side. The rib of the first dorsal vertebra is similar in shape, but its anterior process is vestigial and the ventral grove is lesser. The distal end of the shaft is broken for the two ribs. Other rib fragments were recovered, but their location is uncertain due to their fragmentary nature.

### Appendicular skeleton

#### Humerus

The right humerus was recovered in two fragments (Fig. 6A–B). MMC-894 is the proximal end. The anterior and posterior surfaces of the bone fragment, immediately distally to the proximal articular surface, are greatly roughened. This is consistent with the attachment of strong ligaments. The bone is broken or eroded before the deltopectoral crest, so no description is possible for this structure. MMC-861 is the distal half of the same bone. The cortical bone in both articular surfaces is also eroded, but the general shape can be observed. The articular surface of the distal end shows two condyles and faces caudoventrally (i.e. the distal part of the bone is slightly ventrally curved) (Fig. 6B). The lateral surface of the humerus is slightly convex and the medial slightly concave.

#### Radius

MMC-889 is a slender bone, 12.3 cm long, expanded at both proximal and distal ends, which is interpreted as a left radius (Fig. 6C–D). The distal expansion is fore and aft only whereas the proximal end is both fore and aft and laterally expanded. The shaft is elliptical in cross-section. A soft groove can be observed in the medial side of the proximal end (Fig. 6D).

#### Ilium

The ilium (MMC-838) is very irregular in outline, 10.4 cm high (from posterior ischian process to dorsal blade) and 14.5 cm long (from anterior ischian process to posterior tip of the blade) (Fig. 7A–B). It is extended caudally into a conspicuous posterior process. The anterior end of the blade does not extend cranially than the anterior process which articulates with the ischium. The superior border of the iliac blade is very rounded and wrinkled. The anterior process of the ilium is sharp and extends craniodorsally, near the anterior end of the blade. The posterior process is in a very elevated position. Its external surface is smooth. The posterior tip of the blade is slightly blunt. The ilium has two distinct articulations with the ischium and none for the pubis. Both ischiadic surfaces are situated on stout processes anteriorly separated by a thin wall bone. The anterior ischian process is orientated in a right angle respect to the posterior process. This latter is laterally expanded. The external surface of the ilium is deeply concave between these two ischiadic processes constituting a closed acetabulum. The internal surface of the iliac crest is mainly smooth, but there are wrinkled areas at the superior margin and in the posterior end. The inferior portion of the same internal surface is also occupied by rough surfaces which articulated with the supposed two sacral vertebrae. These areas are separated from each other by a prominent vertical ridge.

#### Femur

The bone (MMC-863) is interpreted as a proximal articular surface of the left femur (Fig. 7C–E). It is craniocaudally flattened (Fig. 7D) and 6.2 cm wide. The articular surface of the head is large, round and very rough. The cranio-medial border near the proximal end is convex (Fig. 7C). The caudal surface is concave, with a ridge in the middle, under the articular surface (Fig. 7E). The bone is broken before the fourth trochanter position.

#### Tibia

The bone (MMC-833) is massive and interpreted as the proximal end of the left tibia (Fig. 7F–G). The articular surface is subtriangular in outline, with the apex of the triangle cranially directed and slightly concave. The proximal portion of the shaft is stout and triangular in section, ornamented with fine longitudinal striations. It is laterally constricted near the anterior side, and it presents a small ridge in its caudolateral side (Fig. 7G). The central portion of the shaft is much more slender than the proximal end. Its section is elliptic.

#### Fibula

They are elongated and slender bones (Fig. 7H–J). The MMC-830 (Fig. 7H, I) is a left proximal end and MMC-844 is a broken shaft (Fig. 7J). The proximal end is expanded and at the same time flattened. In the external side, there is a conspicuous wrinkled area for the attachment of ligaments (Fig. 7H). In the inner side, near the proximal end, there is a smooth surface for the articulation with the tibia showing a rugosity below it within a conspicuous groove (Fig. 7I). The external surface of the partial shaft is smooth, without rugosity. The central portion of the shaft is cylindrical in cross-section.

#### Calcaneum

This bone (MMC-891) extends medially with a very short shaft of 3.8 cm and has an expanded distal end (Fig. 7K–M). This expanded end is very elongated (5.2 cm), robust and conform the larger side of the bone. It contacted with the distal edge of the fibula in its proximal end and the astragalus at the middle of the shaft.

#### Astragalus

MMC-891 is a massive irregular bone 3.2 cm long and 3.4 cm high. The articular surfaces with the tibia and fibula are eroded, but bone is almost complete (Fig. 7N–O), including the fossa for the calcaneum.

#### Phalanges

Two indeterminate phalanges were recovered. MMC-844 is 3.3 cm long and its proximal end is incomplete. It probably is a first phalange. MMC-832 (Fig. 7P–R) is 3.1 cm long and may be a second phalange. It is not possible to elucidate if they belong to the manus or pes.

### Comparison

Cranial remains were compared with species within genus *Allodaposuchus*, hylaeochampsids, *Massaliasuchus*, *Musturzabalsuchus* and *Arenysuchus*. Postcranial bones were compared with available postcranial skeletons of extant crocodylids, alligatorids, gavialids and of the extinct *Diplocynodon*, *Pietraroiasuchus ormezzanoi* and *Isisfordia duncani*.

The external otic region suggests relationship of the specimen from Fumanya (*Allodaposuchus palustris*) with hylaeochampsids and the genus *Allodaposuchus*. The caudal margin of the external otic aperture is different from that of modern crocodylians and most of fossil crocodylomorphs. In the same way as *Hylaeochampsa*, *Allodaposuchus* and *Goniopholis*, the otic aperture of *A. palustris* has not a sharp posterior rim, and the cranioquadrate passage forms the canalis quadratosquamosoexoccipitalis. The quadrate of the studied specimen lacks a dorsal projection medially to the canalis contacting the base of the squamosal at the otic aperture, and the medial margin of the canalis is delimited by the exoccipital. Quadrate foramen aereum is comparatively large than *A*. *subjuniperus*, *A*. *precedens* or *A. subjuniperus.* Both quadrate hemicondyles are more dorsoventrally expanded than in other basal eusuchians such as *A*. *subjuniperus*, *A*. *precedens* or *Hylaeochampsa*. Nevertheless, the hemicondyles do not show the typical expansion observed in most crocodyloids.

The Fumanya specimen shows some skull and jaw characters related to *Allodaposuchus* species. However, it differs in other features from this genus and hylaeochampsids. Unlike other basal eusuchians from the upper Cretaceous of Europe such as *A*. *precedens*, *A*. *subjuniperus*, *Arenysuchus* or *Hylaeochampsa*, there is no shallow fossa in the rostromedial margin of the supratemporal fenestra, being this wall almost vertical. The anterior process of the frontal is much shorter and ornamented compared with *Arenysuchus*, in which it is clearly smooth. Unlike other eusuchians from the upper Cretaceous of Europe, this process in the Fumanya specimen is wider, being its maximum width more than half of the width of the main body of the frontal in the interorbital region. The interorbital region is much narrower than in *A*. *subjuniperus*. The main body of the frontal is slightly concave as in *A*. *precedens* or *Arenysuchus*, but without the sloping present in *Hylaeochampsa* or *Acynodon*, and it is different to the condition presented in *A*. *subjuniperus* whose skull table is totally planar. Frontoparietal suture is concavoconvex in the Fumanya eusuchian. In contrast to *A*. *precedens*, *A*. *subjuniperus* or *Hylaeochampsa*, there is no tubercle or boss on the dorsal surface of the convexity of the paroccipital process of the exoccipital, being totally smooth.

The anterior region of the dentary does not show any festooned outline having the alveolar margin at the same dorsal height, as it has been observed in the genus *Acynodon*. On the contrary, in *Musturzabalsuchus* or in the basal eusuchians from Lo Hueco site in Cuenca (Spain) the dentary is higher at the level of the fourth tooth [21], [28]–[31]. In the Fumanya specimen, *Musturzabalsuchus* and the eusuchians from Lo Hueco the fourth tooth alveolus is the largest, being almost twice as large as the other preserved alveoli. This condition is different in *Acynodon* whose teeth are smaller and similar in size. The tooth morphology resembles to that of *Musturzabalsuchus* [21], [28] and differs from *A*. *precedens*, *A*. *subjuniperus* and *Arenysuchus* [4], [5], [8] whose teeth are longer, with smooth or less ridged surfaces and carinae without false-ziphodont crenulations.

All recovered vertebrae are procoelous, with the exception of the first caudal vertebra that is biconvex. This character clearly indicates that the Fumanya specimen belongs to the clade Eusuchia [19]. The axial skeleton of the Fumanya specimen reveals important differences with extinct and extant taxa, especially in the axis, the first caudal and cervical ribs. The centrum of the axis of the Fumanya eusuchian is completely keeled. This feature clearly differs from other taxa such as *C. acutus*, *C. niloticus*, *O. tetraspis* and *A. mississipiensis* in which only the cranial end of the axis is keeled, or the basal *Isisfordia duncani*, in which the keel is absent. In addition, all of the compared taxa show straight instead of curved centra. The dorsal edge of the neural spine of the axis is horizontally oriented as in *Crocodylus*, *Gavialis* or *Diplocynodon*. In the Fumanya specimen, it is concave in lateral view, without crest, in contrast to all the compared taxa. Complete sutured neural arch and odontoid process suggest an adult specimen.

The preserved presacral vertebrae, except the axis, are similar in shape to those of *C. acutus*, *C. niloticus* and *A. mississipiensis*. Cervical and dorsal vertebrae are similar in size, but the last lumbar and the first caudal are clearly bigger. However, the first caudal is different in shape to all compared taxa. Transverse process of the first caudal is uncommonly shorter and wide.

The cervical rib of the eighth vertebrae is also characteristic. Its articular processes are different in size, length and angle compared to *C. acutus, C. niloticus* and *A. mississipiensis*. These extant taxa show curved instead of straight and symmetrical rib.

The appendicular skeleton seems more conservative in fossil and living taxa. The main differences have been found in the ilium and calcaneum comparing the Fumanya eusuchian with extant taxa. The humerus is similar in shape to those of *C. acutus*, *C. niloticus*, *O. tetraspis. A. mississipiensis*, or *Pietraroiasuchus omezzanoi*. It is similar in size (based on the width of distal condyles) to those of *C. niloticus* and *A. mississipiensis* but scars of the ligaments are more numerous, bigger and conspicuous in *A. palustris*. The radius is slightly bigger but similar in shape to *C. niloticus* and *A. mississipiensis*. However, the medial groove of the radius is not present in any of taxa used for comparison.

The ilium is different in form to *C. acutus*, *C. niloticus*, *O. tetraspis* or *A. mississipiensis*, being more rounded, longer and much taller. However, it is similar in size to the ilium of *A. precedens* reported by Buscalioni *et al.* [7]. The general shape, especially dorsal curvature of the blade, is similar to that of *Diplocynodon* but its caudal tip is not as deep. The posterior tip of the blade of *A. palustris* is similar to that of *Gavialis*. The anterior process is proportionally developed, unlike other compared taxa, including remains assigned to *Allodaposuchus*. The posterior process is stout as in *A. precedens*, but the latter has a blade with straight dorsal margin.

The head of the femur is proportionally larger in *A. palustris* than in *C. niloticus*, or *A. mississipiensis.* It is similar in shape, but the scars of the ligaments are bigger and conspicuous. The tibia is slightly broader and similar in shape to all the other taxa used in comparisons, but the lateral ridge is not seen in any of them, as well as the medial groove of the fibula. The fibula has also conspicuous scars of the ligaments. The calcaneum is larger in *A. palustris* than in the other taxa. Its distal end is uncommonly long, unlike other taxa used in comparisons.

## Phylogenetic Relationships

**Figure 8:**
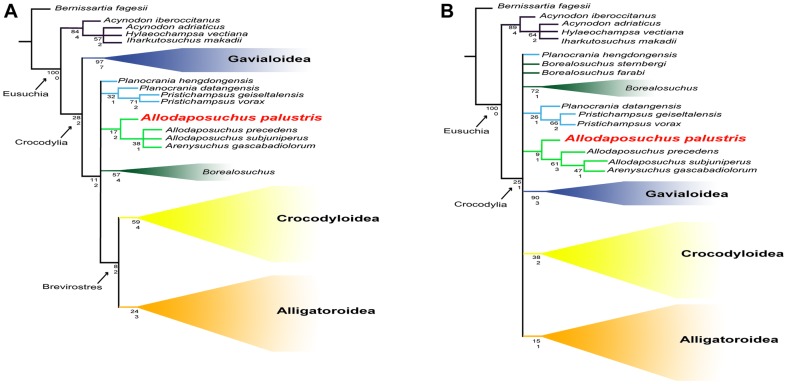
Strict consensus tree of composite data (cranial + postcranial) (left) and cranial only (right). Bootstrap (above) and Bremer (below) values are indicated.

The composite analysis of cranial and postcranial data resulted in 1110 equally parsimonious cladograms of 590 steps (CI  =  0.388; RI  =  0.814; RC  =  0.316). The strict consensus tree topology (Fig. 8A) showed relevant differences with previous works [2]–[9], [32]. In our analysis, the clade (*Allodaposuchus* + *Arenysuchus*) was included within Crocodylia, placed in a more derived position than Gavialoidea, and forming a polytomy with *Borealosuchus*, Planocraniidae and the clade Brevirostres (Crocodyloidea + Alligatoroidea). Nevertheless, this result is consistent with the phylogeny proposed by Puértolas *et al.* [4] in which *Allodaposuchus* is considered the sister taxa of Brevirostres. However in that analysis, *Arenysuchus* and *Allodaposuchus* were not closely related. Relationships within the clade (*Allodaposuchus* + *Arenysuchus*) were weakly resolved (Fig. 8A). There, *A. palustris*, the Fumanya eusuchian, was basal to the clade (*A. precedens* + *A. subjuniperus + Arenysuchus*), which form a polytomy.

To elucidate the effects of postcranial characters in the phylogenetic position of the clade (*Allodaposuchus + Arenysuchus*), the analysis of cranial characters (excluding postcranial data) was also performed (Fig. 8B). It resulted in 1550 equally parsimonious cladograms of 463 steps (CI  =  0.378; RI  =  0.825; RC  =  0.312). In the cranial analysis, the clade (*Allodaposuchus* + *Arenysuchus*) was also placed within Crocodylia but formed a polytomy with planocraniids, *Borealosuchus*, gavialoids, crocodyloids and alligatoroids.

## Taphonomy

**Figure 9:**
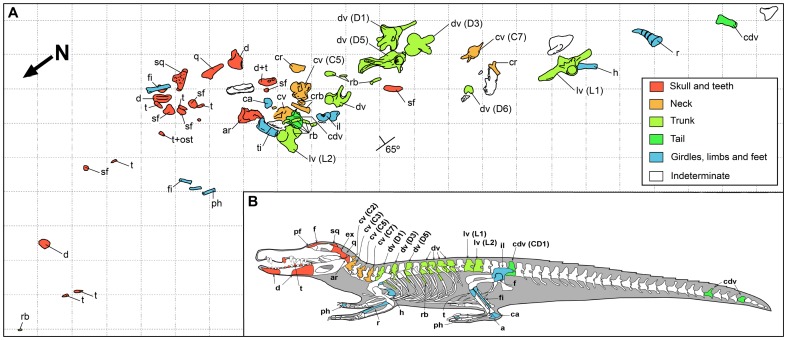
Mapping of the crocodylian bones at the Fumanya Sud locality and their anatomical location. A, distribution of the fossils along a 2.3 m-long accumulation with indication of the type of bone (see legend). Grid square is 10 centimeters. B, anatomical location of the recovered bones. Abbreviations: a, astragalus; ar, articular; ca, calcaneum; cdv, caudal vertebra; cr, cervical rib; cv, cervical vertebra; d, dentary; dr, dorsal rib; dv, dorsal vertebra; eo, exoccipital; ept, ectopterygoid; f, frontal; fi, fibula; fm, femur; h, humerus; il, ilium; lv, lumbar vertebra; ost, osteoderm; pa, parietal; pf, prefrontal; ph, phalanx; q, quadrate; r, radius; rb, rib; sf: skull fragment; sq, squamosal; t, tooth; ti, tibia; ul, ulna.

Remains of *A. palustris* from Fumanya correspond to a 2.3 m-long bone accumulation found within an organic mudstone (Figs. 2 and 9). The bone elements were disarticulated, tightly concentrated in about 1m-long central accumulation with most bones in close contact one to each other, within a centimetric range. In spite of its apparent disarticulation, the bone distribution showed a non-random, anatomical-like arrangement. Thus, the skull fragments and teeth located in the northern edge of the accumulation, the cervical and dorsal vertebrae and ribs in the central part, and some few lumbar and caudal vertebrae in the southern region (Fig. 9). Fragments of the limb bones (phalanges, fibula, femur, tibia, calcaneous) seemed to be randomly distributed throughout the accumulation. Because no duplication of bones belonging to the same side (left or right) existed, we consider the specimen as a single individual.

Bones have no signals of crushing, significant abrasion, weathering or mineralogical crusts nor show superficial scratches or tooth marks. The skull is only represented by partial fragments (quadrates, squamosal, articular, frontal, ectopterygoid, exoccipital, and dentary), which were concentrated together with dentition. Skull fragments exhibit no abrasion. Most of the teeth preserve the enamel, the tip, the root, and delicate and small pseudo-denticles; some teeth are still attached to the enclosing bone. Most of the vertebrae (notably exemplified in MMC-873, MMC-869, MMC-888 and MMC-835) preserve the centrum and parts of the apophyses in connection with the neural arch (Fig. 2D, 5). Limb bones show a variable state of preservation: from partially (humerus, tibia, femur, fibulae) to completely preserved (radius, calcaneous, astragalus, phalanges). The only pelvic element available, the ilium, is completely preserved.

The tight concentration of the bones and the absence of bone sorting suggest an autochthonous accumulation. After death, its body was exposed for a time until the carcass decayed. Bones were probably exposed in the muddy swamp for a time favoring their disarticulation and partial breakage. After that, superficial water currents would have reworked the bones with absent or minimum transport. Final burial of bones was probably produced in the phreatic zone under reducing conditions, as indicated by the organic-matter-rich feature of the embedding black mudstone (after Oms *et al.* [13]). Fossils are black in colour and it probably results from the high hydrocarbon components of the embedded carbonaceous mudstone, a typical trait of swampy environments [33].

The depositional setting of this organic-matter-rich mud is related with a peat-forming environment within a swampy area of an extensive lagoon [13], [17]. In this context, the fossils of *A. palustris* were found in muddy sediments near the area where plant detritus accumulated (i.e., the coal layer about 60 cm below the studied specimen; Fig. 1). Taphonomic evidence suggests that the depositional setting was the same place where the individual inhabited (i.e. swampy areas from coastal wetlands). Although not very common, vertebrate remains have been documented in peat-forming/swampy environments [34], [35]. At Fumanya area, solemydid turtles have been described as inhabitants of related lagoon environments (transitional brackish mudflat [36]).

## Discussion

### Systematics of genus *Allodaposuchus* and the Fumanya eusuchian

The most parsimonious hypothesis obtained in our analyses suggests that the clade (*Allodaposuchus* + *Arenysuchus*) belongs to Crocodylia (Fig. 8). Even though the Bremer and bootstrap values were low, the clade Crocodylia, including (*Allodaposuchus* + *Arenysuchus*), was better supported in the complete analysis (bootstrap  =  28, Bremer index  =  2) than in the cranial analysis (bootstrap  =  25, Bremer index  =  1) (Fig. 8). In this sense, the genus *Allodaposuchus* might represent more derived eusuchian crocodylomorph than previously thought [2], [5], [7], [8], but not as derived as in Martin [9]. This hypothesis is supported by several cranial and vertebral characters. According to Brochu [20], the inclusion of the clade (*Allodaposuchus* + *Arenysuchus*) within Crocodylia would be supported by the following synapomorphies: 1) anterior dentary teeth project anterodorsally, 2) retroarticular process projects posterodorsally, 3) exoccipital lacks boss on paraoccipital process, and 4) hypapophyseal keels are present on the eleventh vertebrae behind the atlas. Absence of the boss in paraoccipital process, only in *A. palustris*, would be an ancestral state reverted in the other members of the clade (*Allodaposuchus* + *Arenysuchus*). The following synapomorphies related *Allodaposuchus* with *Borealosuchus* + Planocraniidae + Brevirostres (*sensu* Brochu [20]): 1) slender postorbital bar, 2) ventral margin of postorbital bar inset from lateral jugal surface, 3) skull table surface planar at maturity, 4) neural arch of the axis lacking lateral processes (diapophyses), 5) wide posterior half of the axis neural spine, 6) axial hypapophysys without deep fork. All these characters except the two former can be observed in *A. palustris*. However, postcranial data are incomplete or lacking for most fossil taxa. Thus, the new phylogenetic location for the genus *Allodaposuchus* should be taken as an alternative hypothesis to be tested with future findings of new complete fossil crocodylomorphs.

Both phylogenetic trees recognized the clade (*Allodaposuchus* + *Arenysuchus*), in which *A. palustris*, the Fumanya eusuchian, was included (Fig. 8). In this sense, the new unranked clade ‘Allodaposuchia’ is proposed. The inclusion of the Fumanya eusuchian within genus *Allodaposuchus* is well supported by the phylogenetic analyses (Fig. 8) and qualitative data. For instance, some skull features of *A. palustris* are found in taxa within genus *Allodaposuchus*: the canalis quadratosquamosoexoccipitalis of the external otic region, quadrate foramen aereum on dorsal surface, the concave surface of the frontal, the margin of the orbits upturned, the squamosal-quadrate suture, the exoccipital between the squamosal and the quadrate posterior to otic aperture, the ventral process of the exoccipital.

*Allodaposuchus palustris* shows some significant differences compared to other members of the clade ‘Allodaposuchia’. The frontal bone and its anterior process are clearly different from *Arenysuchus* in ornamentation, length and concavity (see comparison for details). The interorbital region is much narrower than in *Allodaposuchus subjuniperus*. The Fumanya eusuchian also differs from this latter species and *A. precedens* in the following characters: the surface of the rostromedial margin of the supratemporal fenestra and the elongation of quadrate hemicondyles. The tooth features are very relevant. In *A. palustris*, the teeth are strongly ornamented developing false ziphodont crenulations on the carinae. This ornamentation is lacking in *A. precedens*, *A. subjuniperus* and *Arenysuchus*. All these differences justify the assignment of the specimen from Fumanya to a different species within *Allodaposuchus*.

### Comments on the evolution of postcranial skeleton in eusuchians

Postcranial material is almost unknown for many fossil eusuchians. In the present study, these data were only available for the basal eusuchian *Isisfordia duncani*, the hylaeochampsid *Pietraroiasuchus omezzanoi* and the alligatoroid *Diplocynodon darwinii*. In contrast, postcranial skeletons of several crown taxa were used for comparisons including gavialoids (*Gavialis gangeticus)*, alligatoroids (*Alligator mississipiensis*) and crocodyloids (*Crocodylus niloticus, C. acutus, Osteolaemus tetraspis*). Because of the incompleteness of the database, conclusions regarding to the evolution of postcranial skeleton in eusuchians are preliminary. However, some patterns can be extracted from the available data. In a general view, the postcranial skeletons seem mostly uniform comparing *Allodaposuchus palustris* with taxa within Brevirostres. This is especially evident for most of vertebrae and appendicular skeleton. Major differences were found in the shape of the axis, the first caudal, cervical ribs, the ilium and the calcaneum. These differences might be associated to skull-neck kinesis and locomotion.

### Palaeobiology and palaeoecology of the Fumanya eusuchian

Relatively large size of the lumbar vertebrae, ilium and the astragalus; medial grooves present in the radius and fibula; and more developed attachment of the ligaments in the humerus, femur and fibula suggest a robust crocodile with well-developed musculature. The specimen was probably an adult based on the completely sutured neural arch and odontoid process to the centrum of the axis [37]. According to the measurements of the cranial width and following Verdade [38], the total length inferred for *A. palustris* from Fumanya is 3.72 meters. As it was suggested for *A. precedens* from Oarda de Jos (Romania) [8], the specimen from Fumanya can be considered a generalised predator based on the tooth morphology (robust but pointed teeth) and the absence of bulbous crushing posterior crowns. Following Erickson *et al.* [39], the inferred body mass is of 211 kg and could develop 6788.63 N in its bite force. These magnitudes are similar to those reported [39] for extant typical large crocodylians such as *Crocodylus palustris* Lesson or *Crocodylus intermedius* Graves. For analogy, *A. palustris* might predate small-sized sauropods, which are reported from Fumanya sites [40].

Sedimentology and taphonomy indicate that the habitat of *A. palustris* was a wetland area partially covered by shrubby vegetation (probably abundant cheirolepidiaceous conifers of the genus *Frenelopsis* and a variety of ferns) and tree palms of *Sabalites longirhachis* [17]. The presence of freshwater charophytes and mesohaline (*Saccostrea* oysters) to freshwater (unionids) molluscs indicates changes in the salinity through a set of freshwarer lakes and marshes near the edges of an extensive brackish mudflat [17], [41]. However, related eusuchians from the Maastrichtian of the Pyrenees, such as *Allodaposuchus subjuniperus* and *Arenysuchus gascabadiolorum*, have been found associated to fluvial environments (floodplain deposits laterally close to coastal lagoon), also in the Tremp Formation [4], [5]. *Allodaposuchus precedens* from Oarda de Jos (Romania) was discovered in pond deposits at the base of a series interpreted as a braided fluvial system [8]. This suggests that genera *Allodaposuchus* and *Arenysuchus* were composed of generalist taxa living in both fluvial inner and more coastal lacutrine-palustrine environments.

### Palaeobiogeographic implications

**Figure 10:**
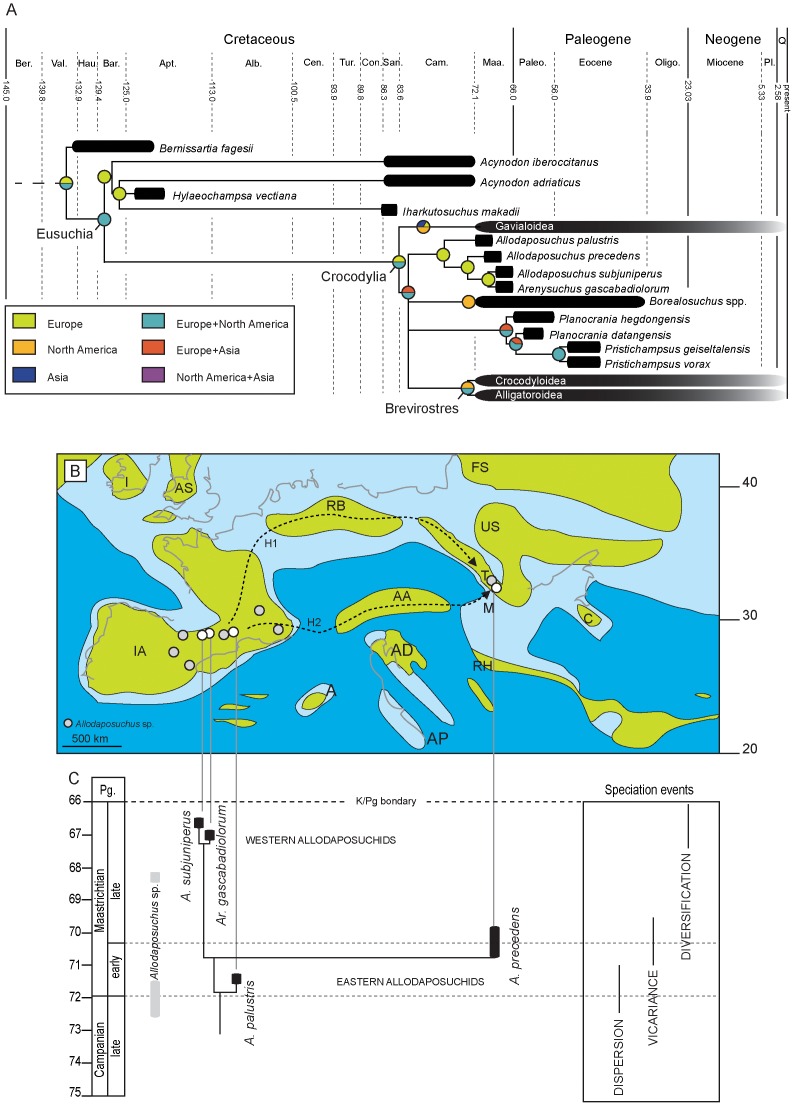
Palaeobiogeographical history of *Allodaposuchus*. A, Time-calibrated phylogram of the Crocodylia based on the phylogenetic hypothesis shown in Fig. 8. The circles at each node represent the relative probabilities for the ancestral areas inferred using the Statistic Divergence-Vicariance Analysis method (S-DIVA). B, Palaeobiogeographical distribution of “Allodaposuchia” showing the two most likely routs for the dispersion of the genus along the European archipelago during the latest Cretaceous. Map modified from Dalla Vecchia [51]. C, Time-calibrated phylogram of “Allodaposuchia” species and their hypothetical speciation events.

It is widely accepted that Eusuchia originated in between Europe and North America during the Early Cretaceous [2], [4], [42], and the same geographical region is proposed as the most likely candidate to host the first members of the order Crocodylia (Fig. 10A). As a whole, the S-DIVA results reported here agree with previous interpretations but also point at a complex early radiation of the clade Crocodylia during the Campanian, involving several dispersal and vicariant events through out the Northern Hemisphere landmass, giving rise to the three main extant superfamilies of crocodiles (Gavialoidea, Crocodyloidea and Alligatoroidea). Because the phylogenetic analyses of the present study cannot resolve the basal polytomy within Crocodylia, there is not a single linage that can unambiguously claim to be the origin of the group. Nevertheless, our results offer a much more complex scenario about the geographical distribution of the first crocodilians than previously thought. If so, first members of Crocodylia would spread through the Northern Hemisphere no later than the Campanian and, by processes of isolation, endemic taxa could rise on different geographical regions, such as *Allodaposuchus* in Europe, or *Borealosuchus* in North America. Less clear it seems to be the ancestral area of the clade Brevirostres, which probably make its firstly apparition somewhere in the Euroamerican region.

During the Late Cretaceous, Europe was divided into a set of islands that formed an archipelago of great palaeobiogeographical and evolutionary interest. Between North America and the European archipelago, palaeogeographical bridges sporadically connected the two continents and permitted faunal exchange between them [43], [44]. Moreover, the layout of Europe as an archipelago fostered endemism and vicariant evolution in terrestrial vertebrates such as crocodylomorphs and dinosaurs [42], [45], [46]. The clade “Allodaposuchia” seems to be endemic of the southern European archipelago where it was widely distributed ―from the Transylvanian domain, at the east, to the Ibero-Armorican domain, at the west― during the Campanian−Maastrichtian (Fig. 9B). Through its geographic and temporal range, the clade diverged into a set of taxa. *Allodaposuchus palustris*, *A. subjuniperus* and *Arenysuchus gascabadiolorum* seem to be endemic of the Ibero-Armorican Island. The presence of *A. precedens* in this island is dubious and based on the specimen MDE/CM-616 studied by Martin [9] and additional fragmentary material. On the contrary, this last species seems endemic the Transylvanian domain [8].

Considering that the oldest remains of the genus *Allodaposuchus* are known from the late Campanian of both Iberian Peninsula [7], [31], [42] and France [7], [47], it seems likely to suggest that this genus could originate elsewhere in the Ibero-Armorican domain, no later than the middle Campanian, and then it dispersed to other European islands. The specimen from Fumanya, together with older findings in the Campanian-Maastrichtian of Armuña (Segovia, Spain) [7], also suggests that the radiation of the genus *Allodaposuchus* might have occurred in wetland environments from the early Maastrichtian of Iberia before a rapid expansion to the remaining archipelago. This is supported by the major diversity of taxa reported in the Ibero-Armorican domain. In this sense, the resulting S-DIVA model suggests a phenomenon of speciation by vicariance for explaining the occurrence of different allodaposuchian species in different European islands, an event that could take place prior to the early-late Maastrichtian boundary (Fig. 10C). This vicariance model may also explain the high degree of endemism of the European faunas. For instance, Weishampel *et al.* [46] postulated that although at high taxonomic level (i.e. family level or higher) Romanian faunas were nearly equal to those from other European regions, but the geographic isolation of the islands during the Late Cretaceous favoured the occurrence of several endemic taxa, such as *Allodaposuchus* species.

Even accepting previous scenario, the route that *Allodaposuchus* could have taken to move from the Ibero-Armorican Island to the Transylvanian one still remains unclear. Given the palaeogeographic configuration and intermittent connection between diverse European islands during the latest Cretaceous (Campanian-Maastrichtian), two dispersal routes arise to be the most likely (Fig. 9B). One possibility is that *Allodaposuchus* could leave the Ibero-Armorican Island taking a northern way to the Renish-Bohemian Island and then move south to the Transylvanian Island. The advantage that this hypothesis offers rests upon the fact that hemipelagic seas separated those islands, with possible intermittent land bridge connection between them. On the other hand, the shortest way for an Ibero-Armorican taxon to reach the Transylanian basins could be that via the Adriatic-Australpine domain. However, according to palaeogeographic reconstructions those areas were separated for eupelagic basins with no possible land connection between them [48]–[50].

Nevertheless, the occurrence of several fossil taxa in the Adriatic-Australpine domain with close phyletic relationship to Ibero-Armorican and Transylvanian faunas, such as dinosaurs [45], [51]–[55], constitutes an irrefutable evidence of the faunal exchange between these areas. How these faunas reached these regions is still matter of debated but “island hopping” dispersion has been proposed [51], [54]. Future findings will shed light on these questions. Finally, with all current phyletic and palaeobiogeographic data at hand, “Allodaposuchia” represents the last truly Europe lineage of crocodilians previous to the end-Cretaceous mass extinction.

## Conclusions

The genus *Allodaposuchus* was traditionally considered a basal eusuchian clade of crocodylomorphs that has historically been comprised of two species (*A. precedens* and *A. subjuniperus*). On the basis of the studied material from the Fumanya Sud locality (southern Pyrenees) we erect the new *Allodaposuchus palustris* species that is diagnosed by the following characters: lack of shallow fossa in the rostromedial margin of the supratemporal fenestra; frontoparietal suture slightly concavoconvex; exoccipital without boss on paraoccipital process; large foramen aereun in quadrate; short and robust teeth with two very marked longitudinal grooves close to the carinae; teeth with strong ornamentation developing false-ziphodont crenulations; anterior process of the ilium more developed. The herein described species add postcranial characters in the cladistical analysis, and provides a new phylogenetic interpretation. Several cranial and vertebral characters are shared with *Borealosuchus* + Planocraniidae + Brevirostres (Crocodyloidea + Alligatoroidea), and suggest a more derived position of *Allodaposuchus* than gavialoids, within Crocodylia. Taphonomic analysis suggests that the studied specimen inhabited coastal wetlands, and thus indicates that the genus *Allodaposuchus* was present in both coastal and fluvial settings. Palaeographical analysis suggests that allodaposuchians diverged in the Ibero-Armorican domain between the middle Campanian and the early Maastrichtian before expanding to the remaining European archipelago.

## Supporting Information

S1 Information **Modifications carried out in operational taxonomic units (OTUs) and characters of the dataset of Brochu, to explore phylogenetic relationships of the specimen from Fumanya Sud locality.**
(DOC)

S2 Information **Modified dataset of Brochu to explore phylogenetic relationships, including both craniodental and postcranial characters.**
(TNT)

S3 Information **Modified dataset of Brochu to explore phylogenetic relationships, including craniodental characters only.**
(TNT)
