# Supplementary material for: Allodaposuchus palustris sp. nov. from the Upper Cretaceous of Fumanya (South-Eastern Pyrenees, Iberian Peninsula): Systematics, Palaeoecology and Palaeobiogeography of the Enigmatic Allodaposuchian Crocodylians
Source: PLoS One. 2014 Dec 31;9(12):e115837. doi: 10.1371/journal.pone.0115837 (PMC4281157; doi:10.1371/journal.pone.0115837)
Supplement: S1 Information — Modifications carried out in operational taxonomic units (OTUs) and characters of the dataset of Brochu, to explore phylogenetic relationships of the specimen from Fumanya Sud locality. (DOC) [file pone.0115837.s001.doc]

**Supporting information S1**

Phylogenetic relationships of the specimen from Fumanya were explored using the dataset of Brochu [1]. However modifications in some operational taxonomic units (OTUs) and characters were carried out.

According to Delfino *et al.* [2], we added a new state to the characters 148 and 149 (102 and 132 in Delfino *et al.* [2]). In both cases, the state 0 is assigned to *Hylaeochampsa*, *Allodaposuchus* and *Arenysuchus*. In the rest of taxa from the original matrix of Brochu [1] the state 0 passes to be 1 and the state 1 becomes 2 (for the description of the new state of character see Delfino *et al.* [2]).

In the present study, *Allodaposuchus precedens* from the original matrix of Brochu [1] was replaced by the codification of the specimen (PSMUBB V 438) from Oarda de Jos (Romania) (see Delfino *et al.*, [2] for details). Brochu [1] used the *Allodaposuchus* cf. *A*. *precedens* (MDE/CM-616, [3]) from France (*Allodaposuchus* sp. in the present paper), filling out the missing characters with the data of other *Allodaposuchus* species from Europe (Brochu, personal communication). Our coding for *A*. *precedens* is the same as in Delfino *et al.* [2] but the characters were reordered according to Brochu [1]. Fifteen new characters that were coded to *A*. *precedens* according to the figures and the description of Delfino *et al.* [2]: 19 (?), 20 (?), 80 (0), 84 (?), 85 (0), 105 (?), 106 (0), 107 (0), 131 (0), 148 (0), 149 (0), 157 (0), 178 (0), 179 (0), 180 (0). In addition, the taxa *Arenysuchus gascabadiolorum* and *Allodaposuchus subjuniperus* were added to the matrix with new codings [4, 5].

The character state codings for *A. palustris* are:

*Allodaposuchus palustris:* ?????????? 0100100??0 1????????? ??1000???? ??????110? ???1?????? ?????????? ??0?????00 ?????????? ?????????? ??????0??? ?????????? ????????0? 0?????1??? ??????0000 0110?1010? ?????????1 ???1??1100 0

**References**

1. Brochu CA (2011) Phylogenetic relationships of *Necrosuchus ionensis* Simpson, 1937 and the early history of caimanines. Zool J Linn Soc 163: S228-S256.
2. Delfino M, Codrea V, Folie A, Dica P, Godefroit P, et al. (2008) A complete skull of *Allodaposuchus precedens* Nopcsa, 1928 (Eusuchia) and a reassessment of the morphology of the taxon based on the Romanian remains. J Vert Paleontol 28: 111-122.
3. Martin JE, Delfino M (2010) Recent advances in the comprehension of the biogeography of Cretaceous European eusuchians. Palaeogeogr, Palaeoclimatol, Palaeoecol 293: 406-418.
4. Puértolas E, Canudo JI, Cruzado-Caballero P (2011) A new Crocodylian from the Late Maastrichtian of Spain: Implications for the initial radiation of crocodyloids. PLoS ONE (6, 6) e20011. doi:10.1371/journal.pone.0020011
5. Puértolas-Pascual E, Canudo JI, Moreno-Azanza M (2013) The eusuchian crocodylomorph *Allodaposuchus subjuniperus* sp. nov., a new species from the latest Cretaceous (upper Maastrichtian) of Spain. Historical Biol. doi:10.1080/08912963.2012.763034
